# Antagonistic effects of activin A and TNF-α on the activation of L929 fibroblast cells via Smad3-independent signaling

**DOI:** 10.1038/s41598-020-77783-8

**Published:** 2020-11-26

**Authors:** Lingling Jiang, Boyang Liu, Yan Qi, Linru Zhu, Xueling Cui, Zhonghui Liu

**Affiliations:** 1 Department of Immunology, College of Basic Medical Sciences, Jilin University, 126 Xinmin Street, Changchun, 130021 Jilin China; 2 Department of General Dentistry, School and Hospital of Stomatology, Jilin University, Changchun, 130021 Jilin China; 3 Department of Genetics, College of Basic Medical Sciences, Jilin University, Changchun, 130021 Jilin China; 4 Department of Scientific Research, Jilin Jianzhu University, Changchun, 130118 Jilin China

**Keywords:** Cell migration, Cell signalling, Cytokines

## Abstract

Fibroblasts play an important role in inflammation and tissue fibrosis. Both activin A and TNF-α can activate immune cells, however, the roles and relationship of them in activating fibroblasts in inflammation remain unclear. Here, this study revealed that TNF-α promoted the release of NO and IL-6 by L929 fibroblast cells, but co-treatment with activin A attenuated these effects. In contrast, activin A induced cell migration and increased the production of tissue fibrosis-related TGF-β1 and fibronectin, while TNF-α inhibited these function changes of L929 cells induced by activin A. Moreover, this study revealed that activin A and TNF-α regulated the activities of L929 cells via ERK1/2/MAPK pathway, rather than Smad3-dependent signaling pathway. Taken together, these data indicate that activin A and TNF-α exert mutually antagonistic effects on regulating fibroblasts activities, and the balance between their action may determine the process and outcome of fibroblasts-mediated inflammation.

## Introduction

Fibroblasts, which are the main cells in loose connective tissue, are the major functional cells during wound healing, and are involved in the regulation of immune response^1^. Fibroblasts exert diverse biological roles in different stages of tissue injury. In the case of the acute inflammation in tissue, some inflammatory mediators, such as nitric oxide (NO), interleukin (IL)-6 and IL-1β, can be released by fibroblasts to participate in the acute response^2,3^. During the post period of inflammation, fibroblasts can proliferate and migrate by themselves, in the meantime of producing extracellular matrix (ECM) like fibronectin (FN) and collagens to promote tissue healing, scar formation and tissue fibrosis^4^.

Tumor necrosis factor-alpha (TNF-α), a vital pro-inflammatory cytokine, can be produced by various cells, including the activated fibroblasts, macrophages, lymphocytes and osteoblasts. TNF-α induces the aggregation and activation of neutrophils and enhances the killing activity of mononuclear macrophages, thus being involved in the acute inflammatory response^5^. Activin A, a member of the transforming growth factor beta (TGF-β) superfamily, is also known as restrictin-P^6^ and plays an important role in numerous processes, such as embryonic development, neuron protection, tumorigenesis and regulation of immunocyte function^7–9^. It has been reported that activin A exerts anti-inflammatory roles in the early inflammation, and participates in tissue repair and fibrosis in the late inflammation^10,11^. Both activin A and TNF-α can activate immune cells to participate in immune response, however, what role TNF-α plays in the process of tissue repair and fibrosis and how it is related to and different from activin A in activating fibroblasts are still unclear.

In this study, we took murine fibroblast cell line L929 as the object to investigate the biological effects of activin A and TNF-α on fibroblasts and the interaction between them in fibroblasts-mediated inflammation.

## Results

### Effects of activin A and TNF-α on NO production in L929 cells

**Figure 1:**
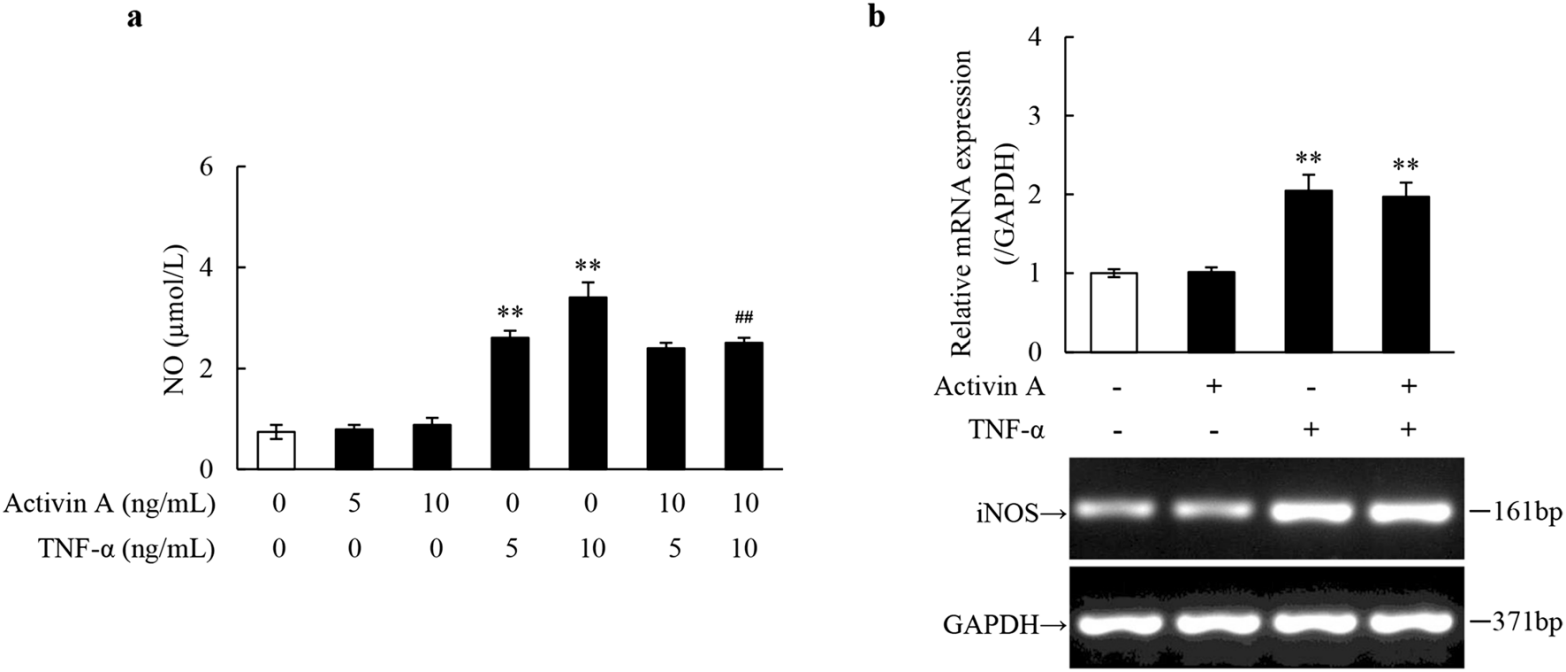
Effects of activin A and TNF-α on NO production in L929 cells. (**a**) The levels of NO were determined in the supernatants of the cultured L929 cells subject to activin A and/or TNF-α at different concentrations for 24 h. (**b**) Expression of iNOS mRNA was examined by RT-PCR in L929 cells subject to 10 ng/mL activin A, 10 ng/mL TNF-α or both for 4 h. The graph represented the relative levels of mRNA expression in three separate experiments. The expression levels of mRNA were normalized against GAPDH expression, and the results were shown as the fold-increase of the control. Full-length gels are presented in Supplementary Fig. 3. *P < 0.05, **P < 0.01 vs. 0 ng/mL control group; ^**##**^*P* < 0.01 vs. TNF-α group with the same concentration.

As a pro-inflammatory cytokine, TNF-α stimulates a variety of cells to secrete inflammatory mediators^12^. Here, this study showed that TNF-α promoted NO secretion by L929 cells. Meanwhile, activin A had no significant effect on NO release by L929 cells, but it inhibited NO secretion by TNF-α-activated L929 cells (Fig. 1a). Moreover, RT-PCR results revealed that TNF-α significantly up-regulated the expression of iNOS mRNA, while activin A had no significant effect on the expression of iNOS mRNA in L929 cells (Fig. 1b). These data suggested that TNF-α promoted NO synthesis and secretion in L929 cells, while activin A had no effect on NO synthesis, which only played a role in regulating NO secretion by TNF-α-activated L929 cells.

### Effects of activin A and TNF-α on IL-6 production in L929 cells

**Figure 2:**
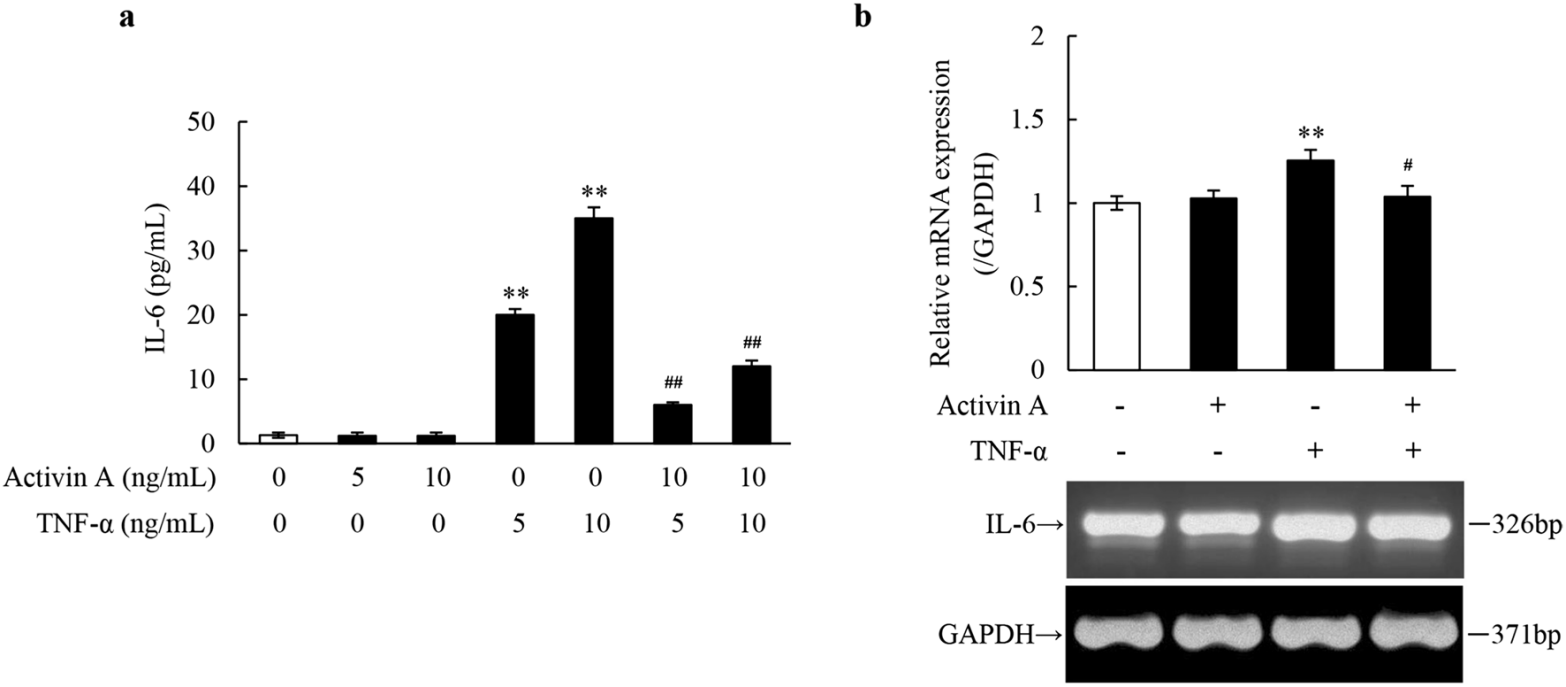
Effects of activin A and TNF-α on IL-6 production in L929 cells. (**a**) The levels of IL-6 were determined in the supernatants of the cultured L929 cells subject to activin A and/or TNF-α at different concentrations for 24 h. (**b**) Expression of IL-6 mRNA was examined by RT-PCR in L929 cells subject to 10 ng/mL activin A, 10 ng/mL TNF-α or both for 4 h. The graph represented the relative levels of mRNA expression in three separate experiments. The expression levels of mRNA were normalized against GAPDH expression, and the results were shown as the fold-increase of the control. Full-length gels are presented in Supplementary Fig. 4. **P < 0.01 vs. 0 ng/mL control group; ^**#**^*P* < 0.05, ^**##**^*P* < 0.01 vs. TNF-α group with the same concentration.

IL-6 is also a pro-inflammatory cytokine generated by fibroblasts, monocytes, lymphocytes and various tumor cells^13^. Therefore, to confirm the antagonistic effect of activin A on the production of pro-inflammatory mediators by TNF-α-activated L929 cells, ELISA was carried out to measure the IL-6 levels in the supernatant of the cultured L929 cells. The results showed that activin A alone had no significant effect on IL-6 production, while TNF-α promoted the release of IL-6 by L929 cells significantly. Moreover, it was shown that the IL-6 levels in L929 cells co-treated with activin A and TNF-α decreased remarkably, compared with that in TNF-α-treated cells (Fig. 2a). Simultaneously, RT-PCR analysis revealed that TNF-α up-regulated the expression of IL-6 mRNA, while activin A down-regulated that in L929 cells co-treated with activin A and TNF-α, even though activin A alone had no significant effect on the expression of IL-6 mRNA in L929 cells (Fig. 2b). These findings indicated that TNF-α might induce the synthesis and secretion of IL-6 in L929 cells, while such effect was inhibited by co-treatment with activin A.

### Effects of activin A and TNF-α on TGF-β1 release by L929 cells

**Figure 3:**
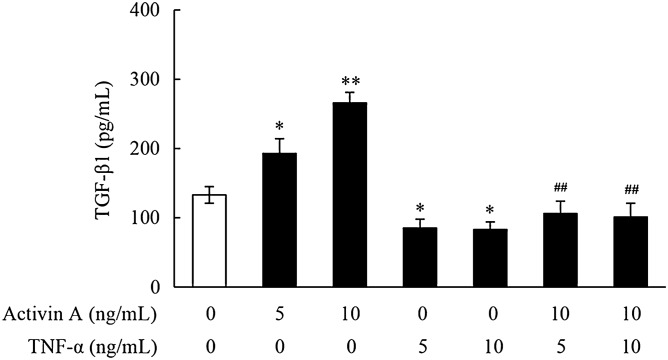
Effects of activin A and TNF-α on release of TGF-β1 in L929 cells. The levels of TGF-β1 were examined by ELISA in the supernatants of the cultured L929 cells subject to activin A and/or TNF-α at different concentrations for 24 h. Data represent mean ± SD (n = 6). *P < 0.05, **P < 0.01 vs. 0 ng/mL control group; ^**##**^*P* < 0.01 vs. activin A group with the same concentration.

TGF-β1 is an important cytokine that mediates tissue fibrosis in chronic inflammation^14^. Therefore, the production of TGF-β1 in L929 cells was further examined. This study revealed that activin A significantly increased the release of TGF-β1 by L929 cells, while TNF-α had opposite effect. Meanwhile, the TGF-β1 production in L929 cells co-treated with activin A and TNF-α remarkably decreased compared with that in activin A-treated cells (Fig. 3). These findings indicated that TNF-α might affect tissue fibrosis through inhibiting activin A-induced TGF-β1 production in fibroblasts.

### Effects of activin A and TNF-α on ECM production of L929 cells

**Figure 4:**
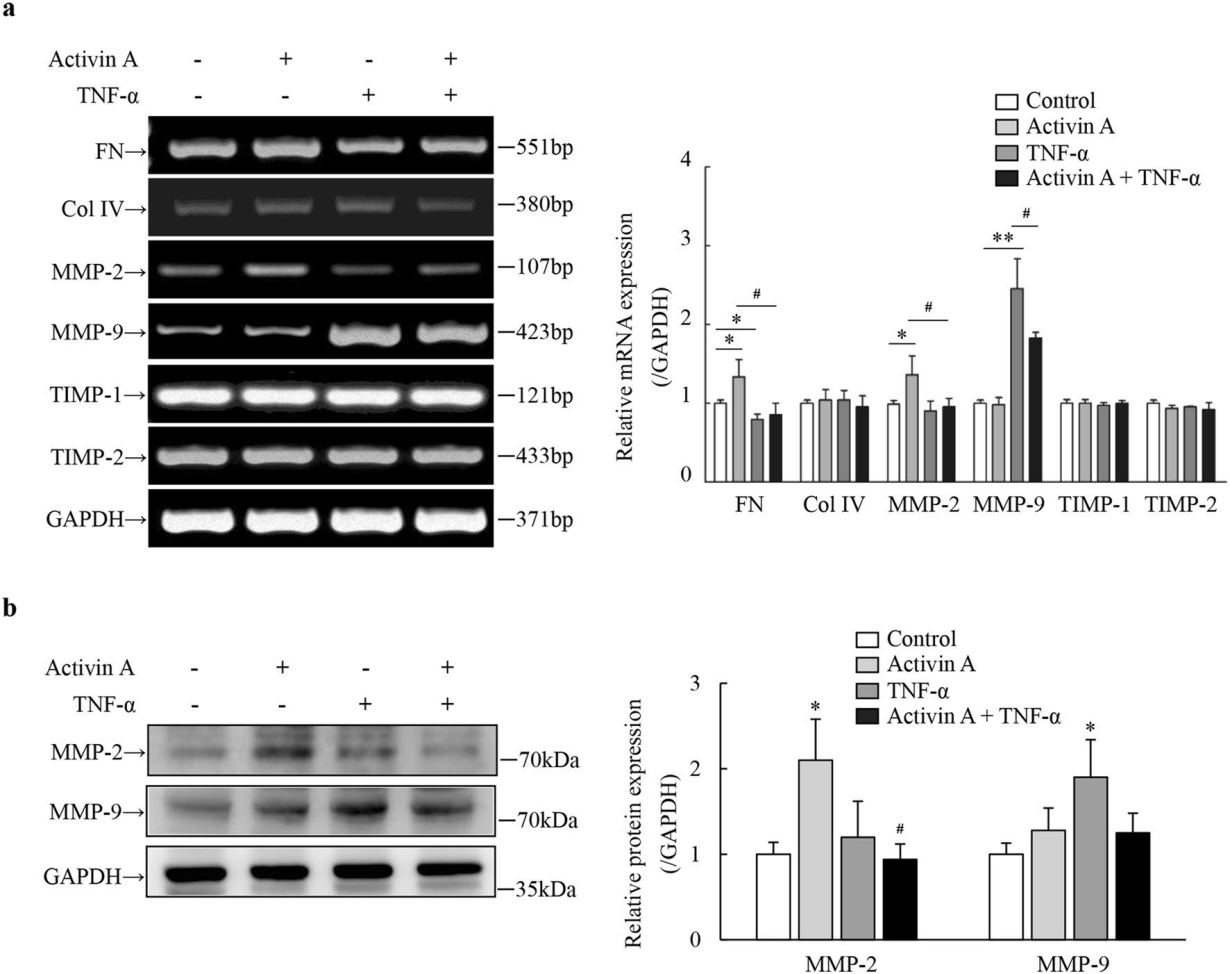
Effects of activin A and TNF-α on ECM production of L929 cells. (**a**) The expression of FN, Col IV, MMP-2, MMP-9, TIMP-1 and TIMP-2 mRNA was examined by RT-PCR in L929 cells subject to 10 ng/mL activin A and/or 10 ng/mL TNF-α for 12 h. The graph represented the relative levels of mRNA expression in three separate experiments. The expression levels of mRNA were normalized against GAPDH expression, and the results were shown as the fold-increase of the control. Full-length gels are presented in Supplementary Fig. 5. (**b**) Levels of MMP-2 and MMP-9 protein expression were examined by Western blotting in L929 cells subject to 10 ng/mL activin A and/or 10 ng/mL TNF-α for 12 h. The graph represented the relative levels of MMP-2 and MMP-9 protein in three separate experiments. The expression levels of these proteins were normalized against GAPDH expression, and the results were shown as the fold-increase of the control. Full-length blots are presented in Supplementary Fig. 6. *P < 0.05, **P < 0.01 vs. control group; ^**#**^*P* < 0.05 vs. activin A group.

To further confirm the involvement of TNF-α in regulation of activin A-mediated tissue fibrosis, the ECM production was examined in L929 cells. As suggested by RT-PCR results, activin A up-regulated the expression of FN and MMP-2 mRNA in L929 cells, but had no effect on MMP-9 mRNA expression. By contrast, TNF-α inhibited the expression of FN mRNA and up-regulated the expression of MMP-9 mRNA. In activin A and TNF-α group, the expression of MMP-2 and FN mRNA were both significantly down-regulated, compared with activin A group, while the expression of MMP-9 mRNA was obviously down-regulated, compared with TNF-α group (Fig. 4a). Moreover, Western blotting results revealed that the expression of MMP-2 protein increased in L929 cells after activin A stimulation, but this effect can be suppressed by TNF-α, as well as the expression of MMP-9 protein was up-regulated significantly in TNF-α-activated L929 cells (Fig. 4b), which was consistent with the results of RT-PCR. These data indicated that activin A was involved in the fibroblasts-mediated tissue fibrosis through inducing TGF-β1 and FN production, while TNF-α might inhibit tissue fibrosis by up-regulating MMP-9 expression and decreasing the production of TGF-β1 and FN in fibroblasts.

### Effects of activin A and TNF-α on migration of L929 cells

**Figure 5:**
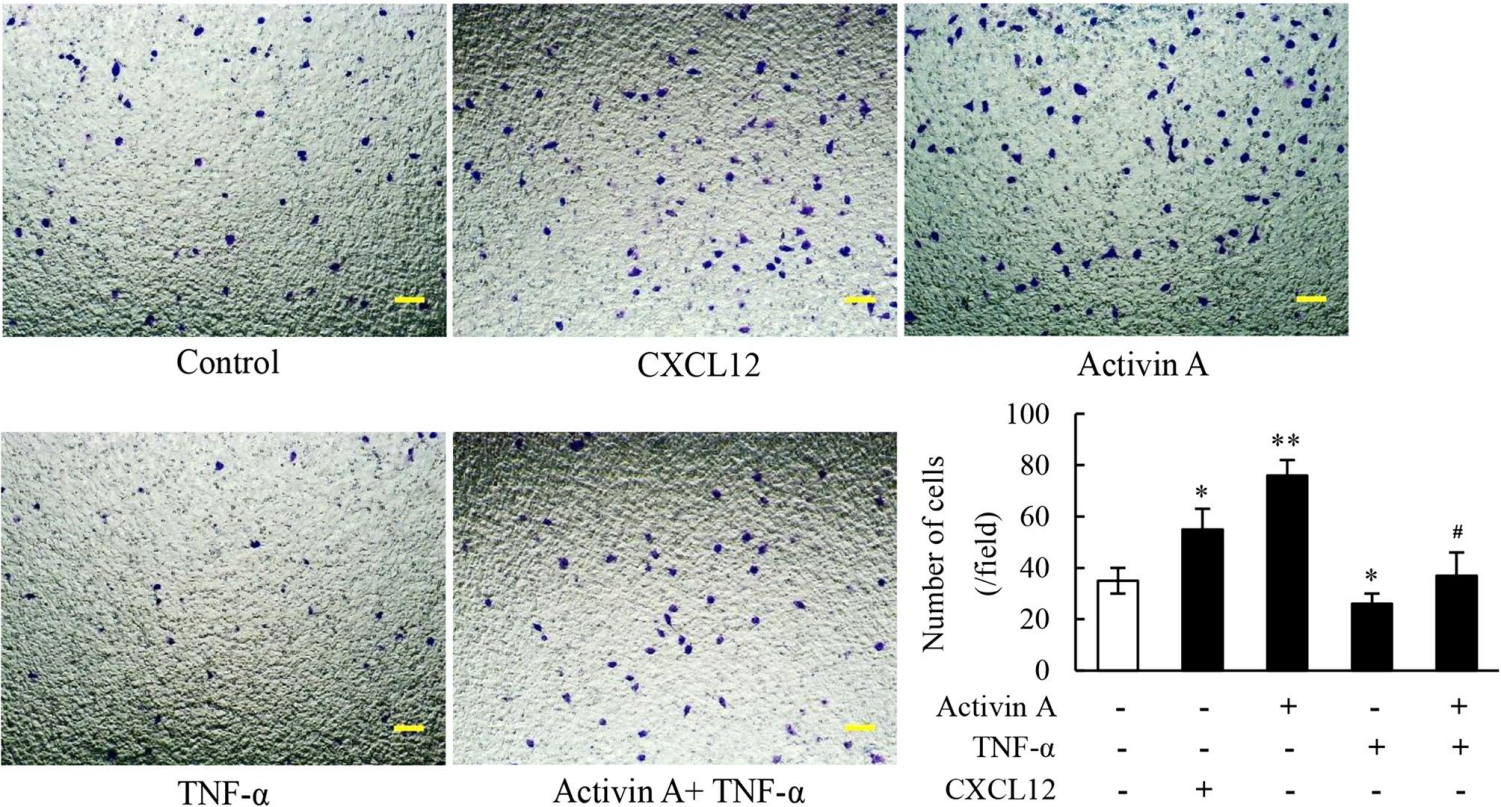
Effects of activin A and TNF-α on migration of L929 cells. The migratory activity of L929 cells treated with 10 ng/mL activin A and/or 10 ng/mL TNF-α was analyzed by Transwell chamber assay. Representative photographs of L929 cells with crystal violet staining are shown. Scale bar = 100 µm. The graph showed the number of crystal violet-stained cells in three separate experiments. *P < 0.05, **P < 0.01 vs. control group; ^**#**^*P* < 0.05 vs. activin A group.

Fibroblasts migration after inflammation response is critical for wound healing and tissue fibrosis. In this regard, the migratory capacity of L929 cells was examined by transwell chamber. The results indicated that both CXCL12 (positive control) and activin A induced the migration of L929 cells, while TNF-α inhibited migration and remarkably suppressed activin A-induced migration of L929 cells (Fig. 5). Based on the results, activin A promoted wound healing and tissue fibrosis in inflammation by inducing fibroblasts migration, while TNF-α might antagonize the above role of activin A.

### Effects of activin A and TNF-α on L929 cells viability

**Figure 6:**
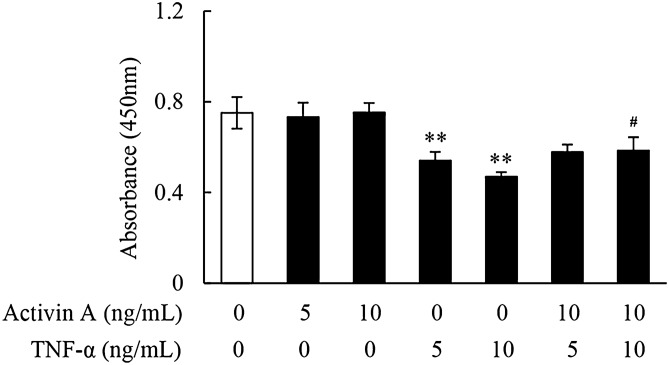
Effects of activin A and TNF-α on L929 cells viability. CCK-8 assay was performed to examine the viability of L929 cells treated with activin A and/or TNF-α at different concentrations for 24 h. The absorbance was measured at 450 nm with a microplate spectrophotometer. Data represent mean ± SD (n = 6). **P < 0.01 vs. 0 ng/mL control group; ^**#**^*P* < 0.05 vs. TNF-α group with the same concentration.

The viability of fibroblasts directly affects the inflammation process. As a result, CCK-8 assay was used to examine cell viability in this study. The data showed that TNF-α inhibited the viability of L929 cells, while activin A had no significant effect on the cell viability, but blocked the inhibitory effect of TNF-α on cell viability (Fig. 6). These above results further demonstrated that TNF-α not only inhibited fibroblasts migration, but also suppressed fibroblasts viability, and might thereby restrain the involvement of fibroblasts in tissue fibrosis.

### Expression of activin receptors, Smads and ERK1/2 in L929 cells

**Figure 7:**
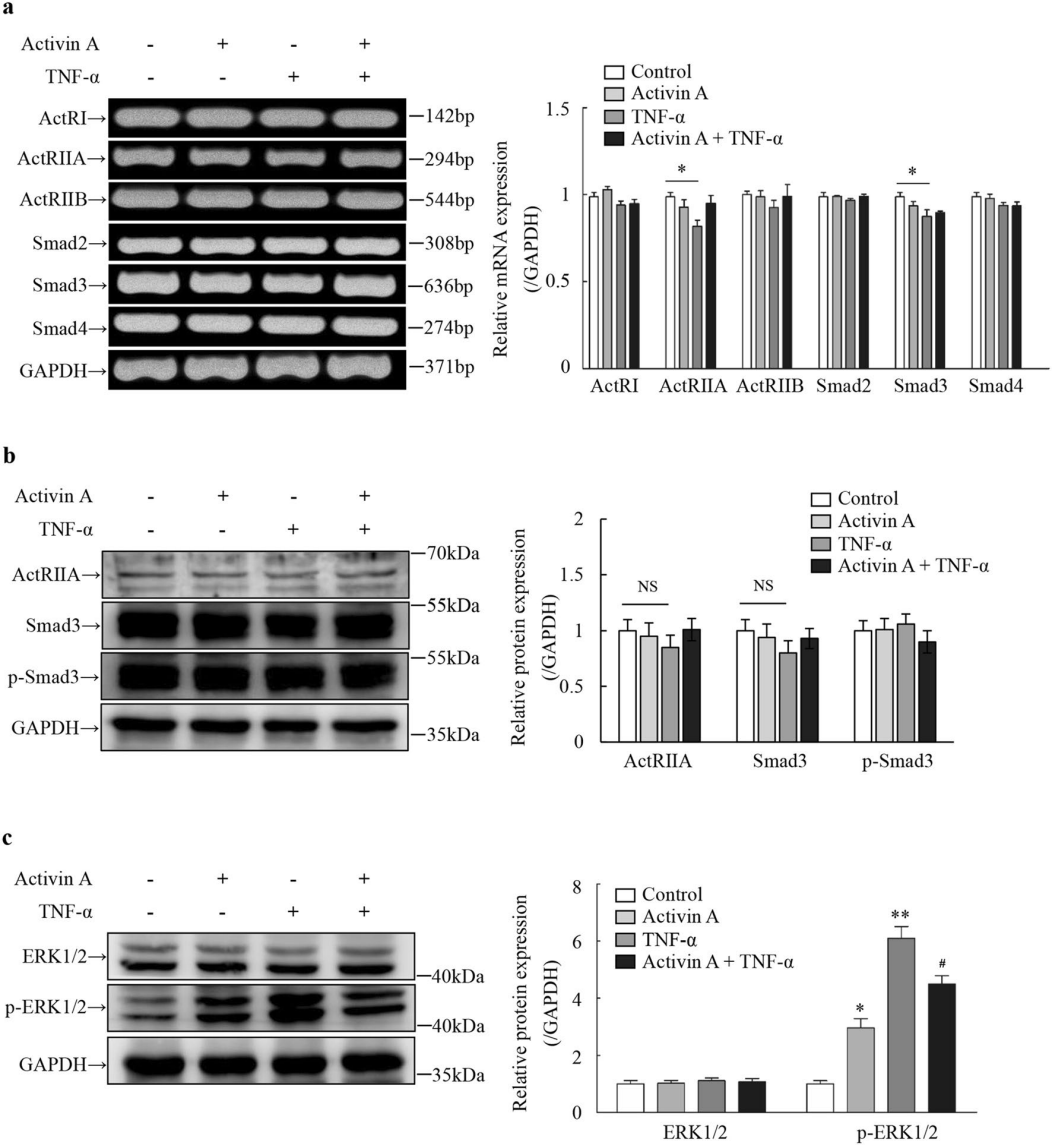
Effects of activin A and TNF-α on expression of activin receptors, Smads and ERK1/2 in L929 cells. (**a**) The expression of activin receptors and Smads mRNA was determined by RT-PCR in L929 cells subject to 10 ng/mL activin A, 10 ng/mL TNF-α or both for 12 h. The graph represented the relative levels of mRNA in three separate experiments. The expression levels of mRNAs were normalized against GAPDH expression, and the results were shown as the fold-increase of the control. Full-length gels are presented in Supplementary Fig. 7. The expression levels of ActRIIA, Smad3, p-Smad3 (**b**), ERK1/2 and p-ERK1/2 (**c**) were examined by Western blotting in L929 cells subject to 10 ng/mL activin A, 10 ng/mL TNF-α or both for 4 h. The graph represented the relative levels of proteins in three separate experiments. The levels of these proteins were normalized against GAPDH expression, and the results were shown as the fold-increase of the control. Full-length blots are presented in Supplementary Fig. 8. NS: not significant; *P < 0.05, **P < 0.01 vs. control group; ^**#**^*P* < 0.05 vs. TNF-α group.

Activin A activates cells via canonical or non-canonical pathways^15^. To determine whether activin A acted on L929 cells via canonical Smad-dependent pathway, the expression of activin receptors and Smads was examined in L929 cells. The RT-PCR results showed that the expression levels of ActRI, ActRIIA, ActRIIB, Smad2, Smad3 and Smad4 mRNA did not change significantly in activin A-treated L929 cells, while TNF-α down-regulated the expression of ActRIIA and Smad3 mRNA (Fig. 7a). Furthermore, Western blotting results revealed activin A had no significant effect on the expression of ActRIIA, Smad3 and p-Smad3, and TNF-α slightly reduced the expression of ActRIIA and Smad3 in L929 cells, but the differences were not statistically significant (Fig. 7b). Moreover, it was also found that both activin A and TNF-α obviously increased the expression of p-ERK1/2, but did not affect the ERK1/2 expression. Meanwhile, activin A inhibited the increase of p-ERK1/2 levels stimulated by TNF-α in L929 cells (Fig. 7c). These data suggested that activin A regulated the activity of L929 cells through ERK1/2/MAPK pathway, rather than Smad-dependent pathway, and activin A might antagonize the biological effects of TNF-α on L929 cells by ERK1/2/MAPK signaling pathway.

## Discussion

As a kind of dimer glycoprotein, activin is composed of two β-subunits of inhibin linked by disulfide. So far, three different forms of activins with the biological activity have been identified, including activin A (βA/βA), activin B (βB/βB) and activin AB (βA/βB)^16^. The three activins have similar biological activities, among which, activin A is the most widely distributed with the highest biological activity. Activin A is highly conserved across species, and its amino acid sequence is 100% conservation between mouse and man^17^. As an important immunoregulator in vivo, activin A exerts effects on the activation of resting immune cells during the onset of inflammation, while at later time points or during the activated cells, activin A inhibits immune response^18^. In the late inflammation, activin A plays a role in injury repair by promoting cell proliferation, differentiation and ECM formation, but the continuous high expression of activin A may induce tissue fibrosis^11^. Activin A expression is up-regulated in the pathological process of various tissue damage and inflammation^19,20^. In addition, activin A is one of the vital cytokines that cause liver, kidney and lung fibrosis^21–23^.

In the process of inflammation, fibroblasts are involved in the early acute response by releasing inflammatory mediators, and they are also one of the important cells related to wound healing and tissue fibrosis, which infiltrate into the damaged site after monocytes/macrophages and neutrophils, gradually replacing white blood cells. Furthermore, fibroblasts can synthesize ECM to repair the damaged tissue and promote tissue fibrosis^24^. TNF-α is a vital inflammatory cytokine, mainly involved in the acute inflammatory response, which is achieved by activating monocytes/macrophages and neutrophils to release inflammatory mediators and proteases^25,26^. During the inflammatory response, the levels of TNF-α and activin A both increase, however, how activin A and TNF-α co-affect fibroblasts activities are still unclear.

In order to determine the roles of activin A and TNF-α in activating fibroblasts, the effects of activin A and TNF-α on the production of inflammatory mediators in L929 cells were first evaluated. Our results showed that TNF-α promoted the synthesis and secretion of NO and IL-6 in L929 cells, while activin A decreased the release of NO and IL-6 by TNF-α-activated cells and inhibited IL-6 synthesis, but had no effect on NO synthesis. Subsequent study further confirmed that TNF-α promoted the secretion of IL-6 in human gingival fibroblasts (HGFs), while activin A suppressed IL-6 secretion by TNF-α-activated fibroblasts (Supplementary Fig. 1). These findings suggest that TNF-α participates in the acute response of inflammation by inducing fibroblasts to synthesize and secrete IL-6 and NO, while activin A may play an anti-inflammatory role in the early inflammation by antagonizing TNF-α action.

Fibroblasts are involved in the process, not only of the acute inflammation, but also of tissue repair and fibrosis in the late inflammation and chronic inflammation^27^. In this case, TGF-β1 plays a critical role in fibrogenesis/fibrosis by regulating the phenotype and function of fibroblasts, such as cell differentiation into myofibroblasts and promoting ECM deposition^28,29^. In the present study, the results showed that activin A promoted the secretion of TGF-β1 in L929 cells, while TNF-α inhibited the production of TGF-β1 induced by activin A. Fibrosis involves a dynamic balance between the biosynthesis and degradation of ECM. FN exhibits numerous biological activities and is related to the formation of tissue fibrosis. MMP-2 and MMP-9 mainly act on type IV collagen, which is extensively distributed in basement membrane and is tightly related to hepatic fibrosis^30^. It has been reported that activin A is involved in the migration and infiltration of macrophages across the basement membrane in inflammatory state by stimulating the production of MMP-2^31^. TIMPs are the natural inhibitors of MMPs, and the interaction between TIMPs and MMPs determine the deposition and degradation of ECM. Our study revealed that activin A promoted the expression of FN and MMP-2 in L929 cells, while co-treatment with TNF-α suppressed this effect. Besides, TNF-α significantly suppressed the expression of FN, but promoted the expression of MMP-9. These findings suggest that activin A promotes fibroblasts-mediated tissue repair and fibrosis in the late inflammation by elevating the production of TGF-β1 and FN, while TNF-α may antagonize activin A-induced tissue repair and fibrosis.

The migration of fibroblasts during inflammation is critical for wound healing and tissue fibrosis. Our previous study has reported that activin A induced the migration of immune cells and tumor cells^32^. In addition, activin A induces the migration and chemotaxis of human periodontal ligament fibroblasts in vitro^33^. In this study, it was demonstrated that both CXCL12 (positive control) and activin A induced L929 cells migration, while TNF-α significantly inhibited L929 cells chemotaxis to activin A. Furthermore, inflammation declines the viability of fibroblasts to hinder the repair and regeneration of tissues, and it has been widely recognized that TNF-α inhibits L929 cells viability^34^, which was also confirmed in this study. Interestedly, activin A antagonized the inhibitory effect of TNF-α on L929 cells viability. This study also verified that activin A existed antagonistic action against TNF-α-induced the decline of cell viability in human gingival fibroblasts (Supplementary Fig. 2). These data further indicate that activin A that is highly expressed in tissue injury and inflammation^35^, may induce fibroblasts migration to local inflammation and improve cell viability to accelerate the process of tissue repair and fibrosis, in contrast, these effects may be blocked by TNF-α, which limits the excessive proliferation and migration of fibroblasts to avoid fibrosis formation.

Activin pathways can be classified into classical Smad-dependent pathway activated by phosphorylation of type I receptor and Smad-independent pathway^36,37^. In the classical Smad pathway, activin A binds to ActRII, which recruits ActRI to form a signal-transducing heterodimer, thus leading to the phosphorylation of cytoplasmic Smad2/3 proteins^38^. Subsequently, the activated Smad2/3 combines with Smad4, which translocates into the nucleus and drives downstream transcriptional targets. Activin A regulates most immune cell activities via Smad-dependent pathway, which is a classic pathway in TGF-β superfamily signaling^39^. Herein, our study showed that L929 cells expressed activin receptors and Smads. But activin A did not affect the expression of ActRI, ActRII and Smad2,3,4 mRNA, and both activin A and TNF-α did not alter the levels of ActRIIA, Smad3 and p-Smad3 proteins in L929 cells. It is suggested that activin A and TNF-α might not regulate the activity of L929 cells via Smad-dependent pathway.

In addition to the Smad-dependent pathway, activin A also regulates the biological activity of cells by other pathways, among which, MAPKs pathway has been reported to be associated with the action of activins and TNF-α^40,41^. MAPKs belong to the highly conserved serine/threonine protein kinase family, which are involved in regulating numerous cell processes, including proliferation, differentiation, survival, apoptosis, cell stress and inflammatory response^42^. MAPKs are activated by a conserved signaling cascade from yeast to mammals^43^. At the present, three parallel MAPK signaling pathways have been identified in mammals, which are ERK signaling, JNK signaling and p38 MAPK signaling pathways^44^. It has been reported that the individual activation of ERK2 and JNK1 pathways contributes to TNF-α-induced IL-8 expression in synovial fibroblasts^45^. In this study, we found that both activin A and TNF-α increased the levels of p-ERK1/2 protein, and activin A inhibited the increase of p-ERK1/2 in TNF-α-activated L929 cells. These results suggest that both activin A and TNF-α regulate the activities of L929 cells via ERK1/2/MAPK pathway, and activin A may antagonize the biological effect of TNF-α on L929 cells by ERK1/2/MAPK signaling. Further studies of the specific pathways, by which activin A and TNF-α antagonize each other in L929 cells activation, are required to elucidate.

Previous studies have reported that activin A elicits an anti-inflammatory role in regulating the activation of various immune cells^46,47^. In the present study, it was further revealed that activin A inhibited TNF-α-mediated inflammation in fibroblasts, which confirmed the critical role of activin A in inflammation regulation. However, our study has some limitations due to using a murine cell line in vitro. Therefore, we further utilized human gingival fibroblasts to verify the effects of activin A in the text of TNF-α-mediated inflammation. Results showed activin A had similar action in human fibroblasts as in mouse fibroblasts, which elucidated that this discovery had potential clinical application value. Moreover, our data revealed TNF-α antagonized fibroblasts-mediated fibrosis induced by activin A, which might be an important factor to avoid tissue fibrosis in the late inflammation. Currently, we are further investigating the effects and mechanism of activin A and TNF-α on the activation of human fibroblasts to provide a novel idea and theoretical basis for clinical inflammation treatment.

In summary, these above results indicate that activin A plays an anti-inflammatory role in TNF-α-activated fibroblasts, while TNF-α exerts an anti-fibrotic role in activin A-activated fibroblasts. Therefore, these findings suggest that activin A and TNF-α have mutually antagonistic effects on the activation of fibroblasts, and the interaction between them may determine the process and outcome of fibroblasts-mediated inflammation.

## Materials and methods

### Cell culture

Murine fibroblast cell line L929 cells (American Type Culture Collection, ATCC, Rockville, MD, USA) were maintained in high glucose DMEM supplemented with 10% fetal bovine serum (FBS) in a moist atmosphere at 37 °C with 5% CO_2_.

### Reagents

Recombinant human/mouse/rat activin A was provided by R&D systems (Minneapolis, MN, USA). Recombinant murine TNF-α and recombinant murine SDF-1α (CXCL12) were obtained by PeproTech (Rocky Hill, NJ, USA). Cell Counting Kit-8 (CCK-8) was bought from GlpBio Biotechnology Co. (Shanghai, China). NO detection kit was purchased Beyotime Biotechnology Co. (Shanghai, China). Enzyme-linked immunosorbent assay (ELISA) kit for TGF-β1 was obtained from eBioscience (San Diego, USA); ELISA kit for IL-6 was provided by R&D systems (Minneapolis, MN, USA). Reverse transcription-PCR (RT-PCR) kit was purchased from Takara Biotechnology Co. (Dalian, China). The antibodies used for Western blotting were as follow: ActRIIA (Absin), Smad3 (Immunoway), p-Smad3 (Abcam), ERK1/2 and p-ERK1/2 (Cell Signaling), MMP-2 (Absin), MMP-9 (Absin) and GAPDH (Absin).

### Cell viability assay

Cell viability was determined using CCK-8 assay. L929 cells (2 × 10^4^ cells per well) were seeded into a 96-well plate and incubated in 1% FBS-DMEM containing activin A (0, 5 and 10 ng/mL), TNF-α (5 and 10 ng/mL) and activin A (10 ng/mL) plus TNF-α (5 or 10 ng/mL) at 37 °C in 5% CO_2_ for 24 h, respectively. 10 μL of CCK-8 reagent was added into the culture medium of each well, and the cells were incubated for 2 h at 37 °C. The absorbance was detected at 450 nm with a microplate spectrophotometer. Each experiment was carried out in triplicate.

### Griess assay

NO level was determined by assaying the accumulation of nitrite, the primary stable breakdown product of NO in the supernatants of the cultured cells with the Griess reagent^48^. Briefly, 50 μL of the culture supernatant was transferred to 96-well plates and incubated with 50 μL of Griess reagent I for 10 min at room temperature in the dark, and followed by incubation with 50 μL of Griess reagent II for 10 min at room temperature in the dark according to the manufacturer’s protocol. The absorbance of samples was measured at 540 nm using a microplate spectrophotometer. The dilution series of sodium nitrite (0–100 μM) was used to generate the nitrite standard reference curve.

### Enzyme-linked immunosorbent assay

The supernatants of the cultured L929 cells were collected and the levels of IL-6 and TGF-β1 were determined by ELISA kits according to the manufacturer’s protocol. The absorbance was then detected at 450 nm to evaluate the IL-6 or TGF-β1 levels using a microplate spectrophotometer.

### Transwell chamber assay

Migration of L929 cells was examined using a Transwell chamber as described previously^49^. Briefly, L929 cells (2 × 10^4^ cells in 200 μL culture medium with 1%FBS) were placed upon the cell culture inserts (8 μm pore size; Corning, NY, USA). The lower chambers were filled with 500 μL culture medium containing activin A (0 and 10 ng/mL), TNF-α (10 ng/mL), activin A (10 ng/mL) plus TNF-α (10 ng/mL) or CXCL12 (100 ng/mL) as positive control. After incubated for 10 h, the cells on the upper side of the insert were removed with cotton-tipped swabs. The cells that had passed through the insert membranes were fixed with 4% paraformaldehyde for 10 min and stained with 0.1% crystal violet. The stained cells were imaged on an inverted microscope and cells numbers were counted in five randomly chosen fields from each chamber.

### RT-PCR

Total RNA was extracted from L929 cells using TRIzol reagent (Takara, Dalian, China) according to manufacturer’s instructions. The 1 μg total RNA was reverse-transcribed into cDNA using the cDNA Synthesis Kit (Takara, Dalian, China). PCR was performed using PCR kit (Takara, Dalian, China) under the following conditions: 95 °C for 90 s, followed by 35 cycles at 94 °C for 30 s, 55–60 °C for 20 s and 72 °C for 40 s, followed by a final 10 min extension step at 72 °C. PCR products were separated by 2% agarose gel electrophoresis and stained with Super Gelred (US EVERBRIGHT, Suzhou, China). The specific bands were analyzed using Image J software and the expression levels of these genes were normalized against GAPDH^50^. Primers sequences were available upon Table 1.

**Table 1:** Primer sequences for RT-PCR.

Gene	Primer	Sequence (5′–3′)	Fragment size (bp)	Tm (℃)
GAPDH	F	GATTGTTGCCATCAACGACC	371	56
	R	GTGCAGGATGCATTGCTGAC		
ActRI	F	GGTGTAACAGGAACATCACGG	142	56
	R	GCAACTCCAAGGATGCAAGCT		
ActRIIA	F	ATTGGCCAGCATCCATCTCTTG	294	56
	R	GCCACCATCATAGACTAGATTC		
ActRIIB	F	TGCTGAAGAGCGACCTCAC	544	56
	R	AGCAGGTCCACATTGGTGAC		
Smad2	F	ATGGCCGTCTTCAGGTTTCACA	308	56
	R	ACTCTGTGGCTCAATTCCTGCT		
Smad3	F	CCAGCACACAATAACTTGGA	636	56
	R	AGACACACTGGAACAGCGGA		
Smad4	F	GAGGTGGCCTGATCTACACA	274	56
	R	TGATGCGCGATTACTTGGCG		
iNOS	F	ATGGCAACATCAGGTCGG	161	56
	R	GCACAACTGGGTGAACTCC		
IL-6	F	ACTGATGCTGGTGACAACCAC	326	56
	R	CCAGGTAGCTATGGTACTCCA		
FN	F	TGGCTGTCAGTCAGAGCAAG	551	56
	R	GGTACAGGTGATGCGTCCAT		
Collagen IV	F	GCCTGCTCAAGGAGAAGACA	380	54
	R	GATCCATAGGAGTCTCCAGGT		
MMP-2	F	GCCTCATACACAGCGTCAATCTT	107	56
	R	CGGTTTATTTGGCGGACAGT		
MMP-9	F	GCCCTACAGCGCCCCCTACT	423	56
	R	AGACACGCCCCTTGCTGAACA		
TIMP1	F	ACTGGAGAGTGACACTCACTG	121	56
	R	AAGGGATCTCCAGGTGCACAA		
TIMP2	F	GCAATGCAGACGTAGTGATCAG	433	56
	R	ATGCTCTTCTCTGTGACCCAGT		

### Western blotting

Western blotting of proteins was performed as described previously^51^. Briefly, L929 cells were lysed using protein extraction reagent (Thermo Scientific, USA) containing protease & phosphatase inhibitor cocktail (Thermo Scientific, USA) and 0.5 M EDTA solution. Then the lysates were centrifuged at 12,000 × *g* at 4 ℃ for 30 min, and the proteins were quantified using the BCA protein assay kit (Thermo Scientific, USA) following the manufacturer’s instruction. 30 μg of proteins were separated by electrophoresis with 10% SDS-PAGE gel, and transferred onto a polyvinylidene difluoride membrane. The membranes were blocked in 5%BSA-TBS for 2 h at RT, and then incubated with the specific antibodies overnight at 4 ℃. The membranes were further incubated with a corresponding horseradish peroxidase-conjugated second antibodies for 1 h at RT. Finally, the labeled proteins were detected by chemiluminescence (GE Healthcare, UK). The immunoblots were quantified using Image J software and the expression levels of these proteins were normalized against GAPDH.

### Statistical analysis

All data are expressed as mean ± standard deviation (SD). Analyses were performed using GraphPad Prism 5 software. Statistical significance was determined by one-way analysis of variance (ANOVA). A probability value of *P* < 0.05 was considered statistically significant.

## Supplementary information

Supplementary Information.
