# Supplementary material for: Antagonistic effects of activin A and TNF-α on the activation of L929 fibroblast cells via Smad3-independent signaling
Source: Sci Rep. 2020 Nov 26;10:20623. doi: 10.1038/s41598-020-77783-8 (PMC7693280; doi:10.1038/s41598-020-77783-8)
Supplement: Supplementary file 1 — Supplementary Information. [file 41598_2020_77783_MOESM1_ESM.pdf]

Supplementary Information

Antagonistic effects of activin A and TNF-α on the activation of L929 fibroblast cells via Smad3-independent signaling

Lingling Jiang[1,2], Boyang Liu[3,4], Yan Qi[1], Linru Zhu[1], Xueling Cui[3] & Zhonghui Liu[1*]

[1]Department of Immunology, College of Basic Medical Sciences, Jilin University, Changchun, Jilin 130021, China. [2]Department of General Dentistry, School and Hospital of Stomatology, Jilin University, Changchun, Jilin 130021, China. [3]Department of Genetics, College of Basic Medical Sciences, Jilin University, Changchun, Jilin 130021, China. [4]Department of Scientific Research, Jilin Jianzhu University, Changchun, Jilin 130118, China.

*Correspondence and requests for materials should be addressed to Z.L. (email: liuzh@jlu.edu.cn)

a **b**

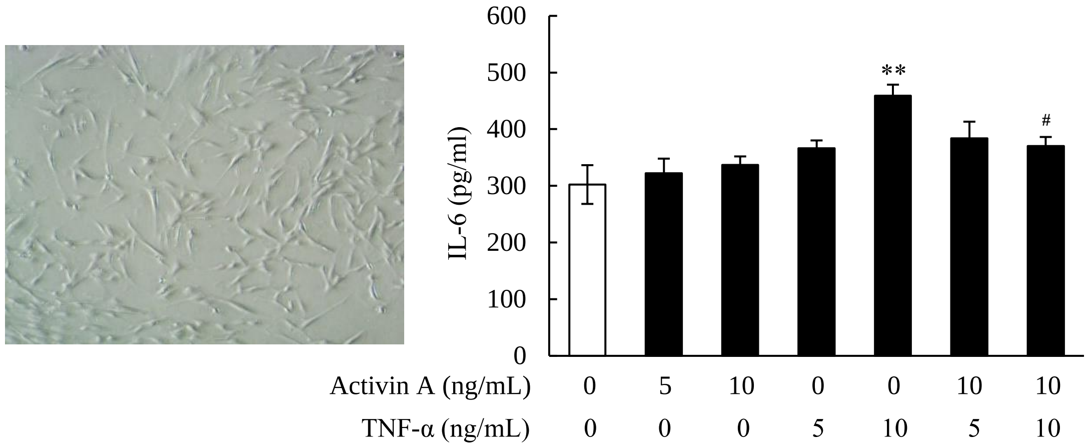

Supplementary Figure 1. Effects of activin A and TNF-α on IL-6 production by HGFs. (**a**) The primary cultured human gingival fibroblasts (HGFs) were shown (40×). (**b**)The levels of IL-6 were determined in the supernatants of the cultured HGFs treated with activin A, TNF-α or both for 24h. Data represent mean ± SD (n=6). ***P*<0.01 vs. 0 ng/mL control group; #*P*<0.05 vs. TNF-α group with the same concentration.

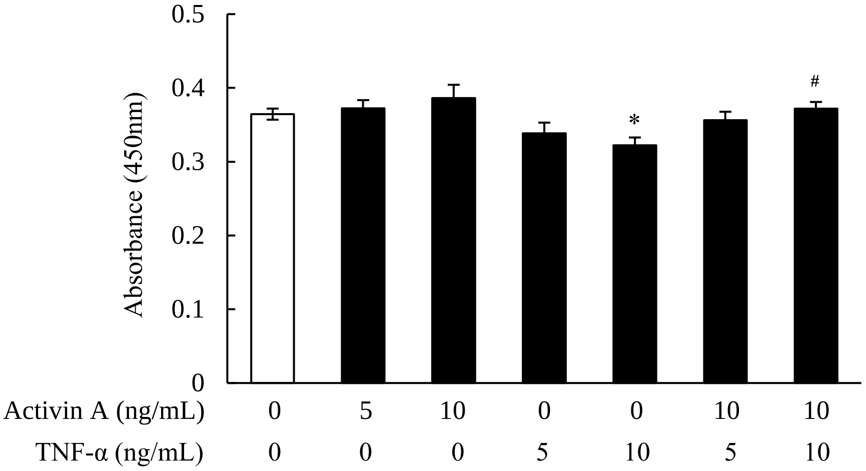

Supplementary Figure 2. Effects of activin A and TNF-α on the viability of HGFs. CCK-8 assay was used to examine the viability of HGFs treated with activin A and/or TNF-α for 24h. The absorbance was measured at 450nm with a microplate spectrophotometer. Data represent mean ± SD (n=6). *P<0.05 vs. 0 ng/mL control group; #P<0.05 vs. TNF-α group with the same concentration.

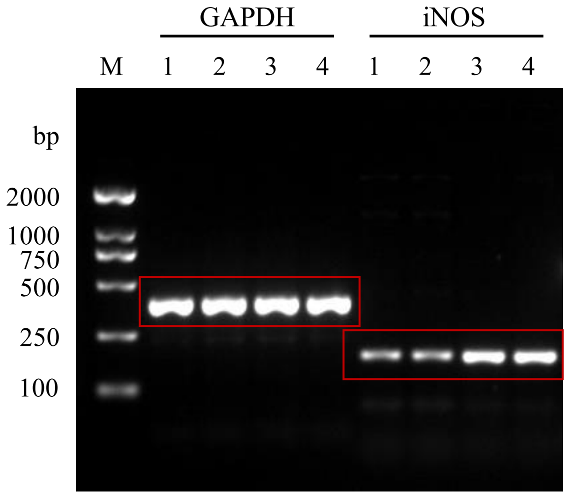

Supplementary Figure 3. Full-length gels of cropped images (red boxes) shown in manuscript Figure 1b. M, molecular weight marker (bp); Lane 1, control; Lane 2, activin A (10 ng/ml); Lane 3, TNF-α (10 ng/ml); Lane 4, activin A (10 ng/ml) + TNF-α (10 ng/ml).

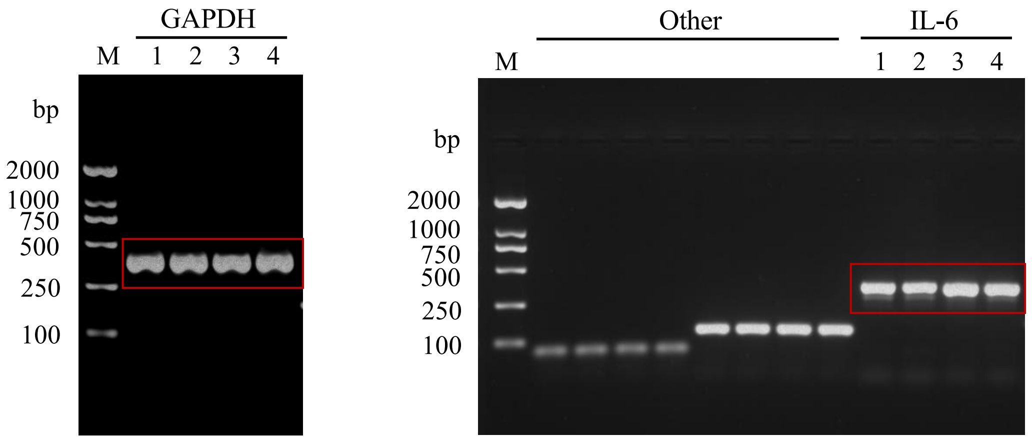

Supplementary Figure 4. Full-length gels of cropped images (red boxes) shown in manuscript Figure 2b. M, molecular weight marker (bp); Lane 1, control; Lane 2, activin A (10 ng/ml); Lane 3, TNF-α (10 ng/ml); Lane 4, activin A (10 ng/ml) + TNF-α (10 ng/ml).

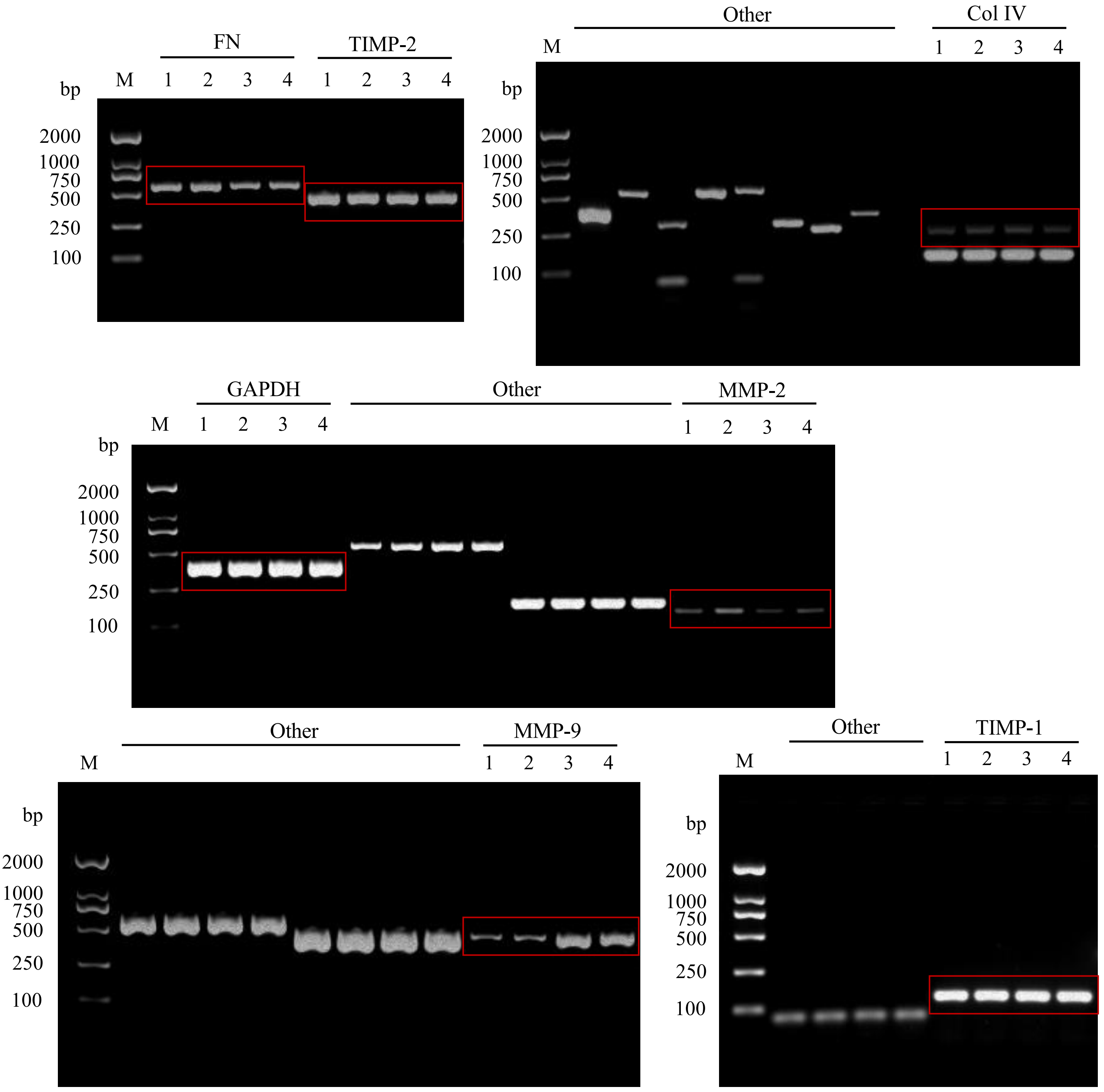

Supplementary Figure 5. Full-length gels of cropped images (red boxes) shown in manuscript Figure 4a. M, molecular weight marker (bp); Lane 1, control; Lane 2, activin A (10 ng/ml); Lane 3, TNF-α (10 ng/ml); Lane 4, activin A (10 ng/ml) + TNF-α (10 ng/ml).

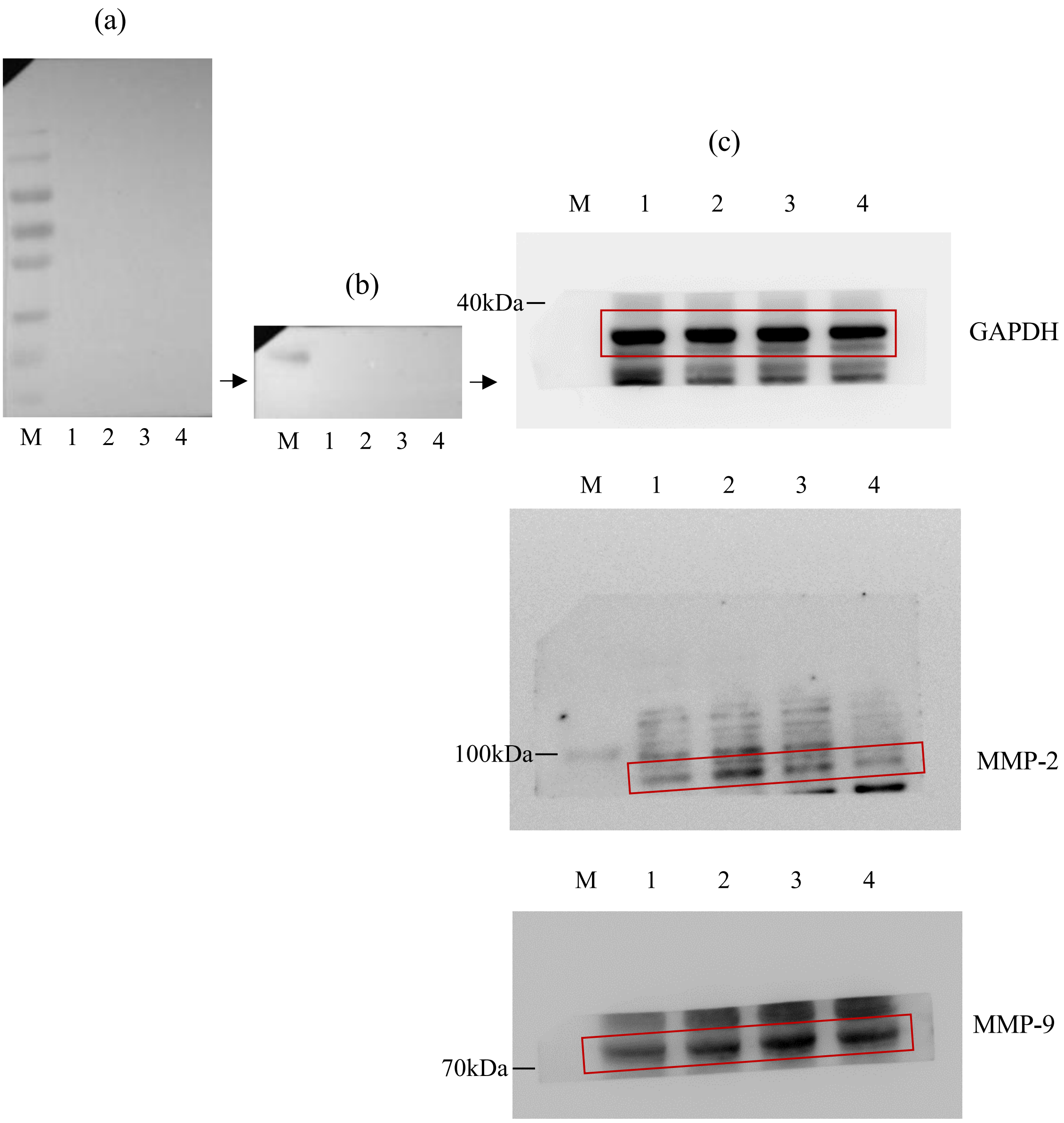

Supplementary Figure 6. Full-length blots of cropped images (red boxes) shown in manuscript Figure 4b. The membrane of full-length western blots (a) was cut into (b) size according to colorful molecular marker of protein, and then the (b) size membranes were probed with anti-GAPDH, anti-MMP-2 or anti-MMP-9 antibodies and the labeled proteins were detected by chemiluminescence (c). M, colorful molecular marker of protein. Lane 1, control; Lane 2, activin A (10 ng/ml); Lane 3, TNF-α (10 ng/ml); Lane 4, activin A (10 ng/ml) + TNF-α (10 ng/ml).

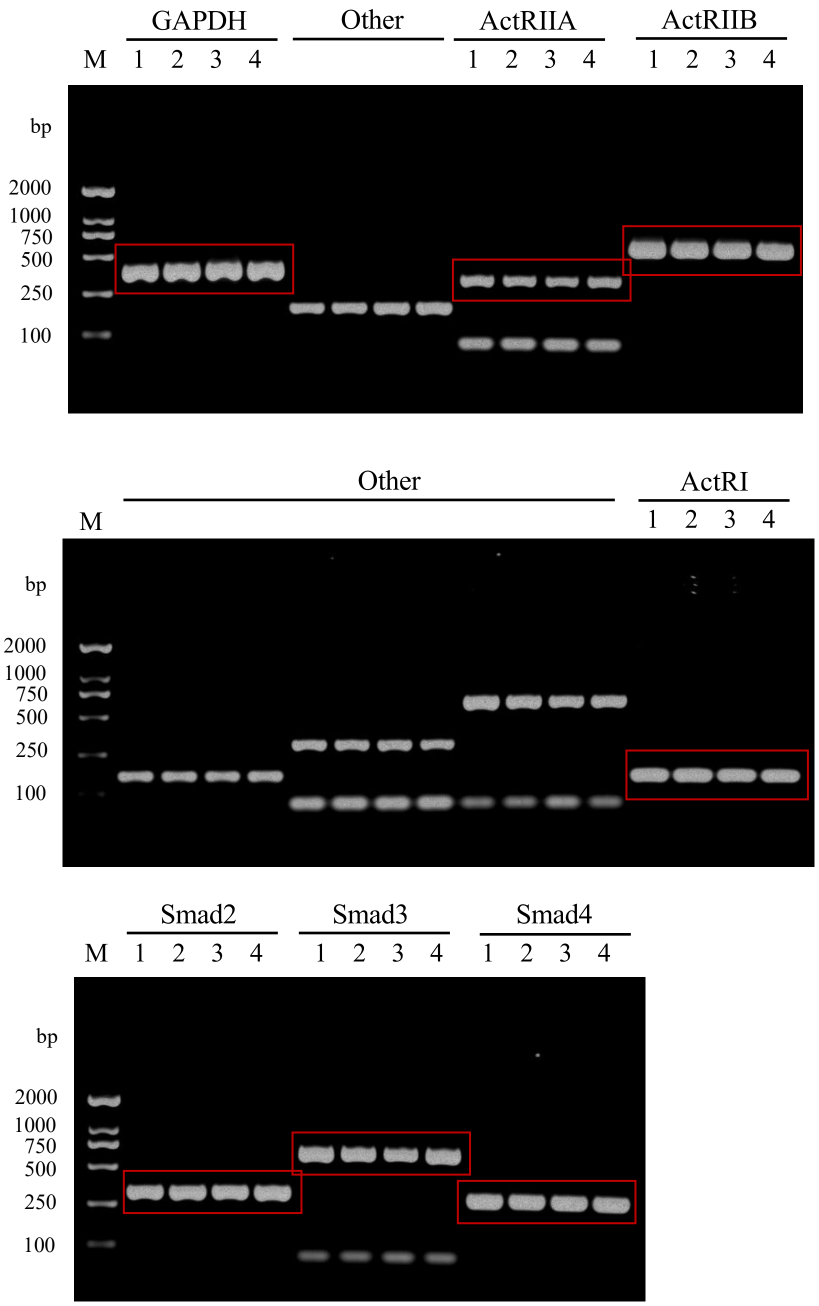

Supplementary Figure 7. Full-length gels of cropped images (red boxes) shown in manuscript Figure 7a. M, molecular weight marker (bp); Lane 1, control; Lane 2, activin A (10 ng/ml); Lane 3, TNF-α (10 ng/ml); Lane 4, activin A (10 ng/ml) + TNF-α (10 ng/ml).

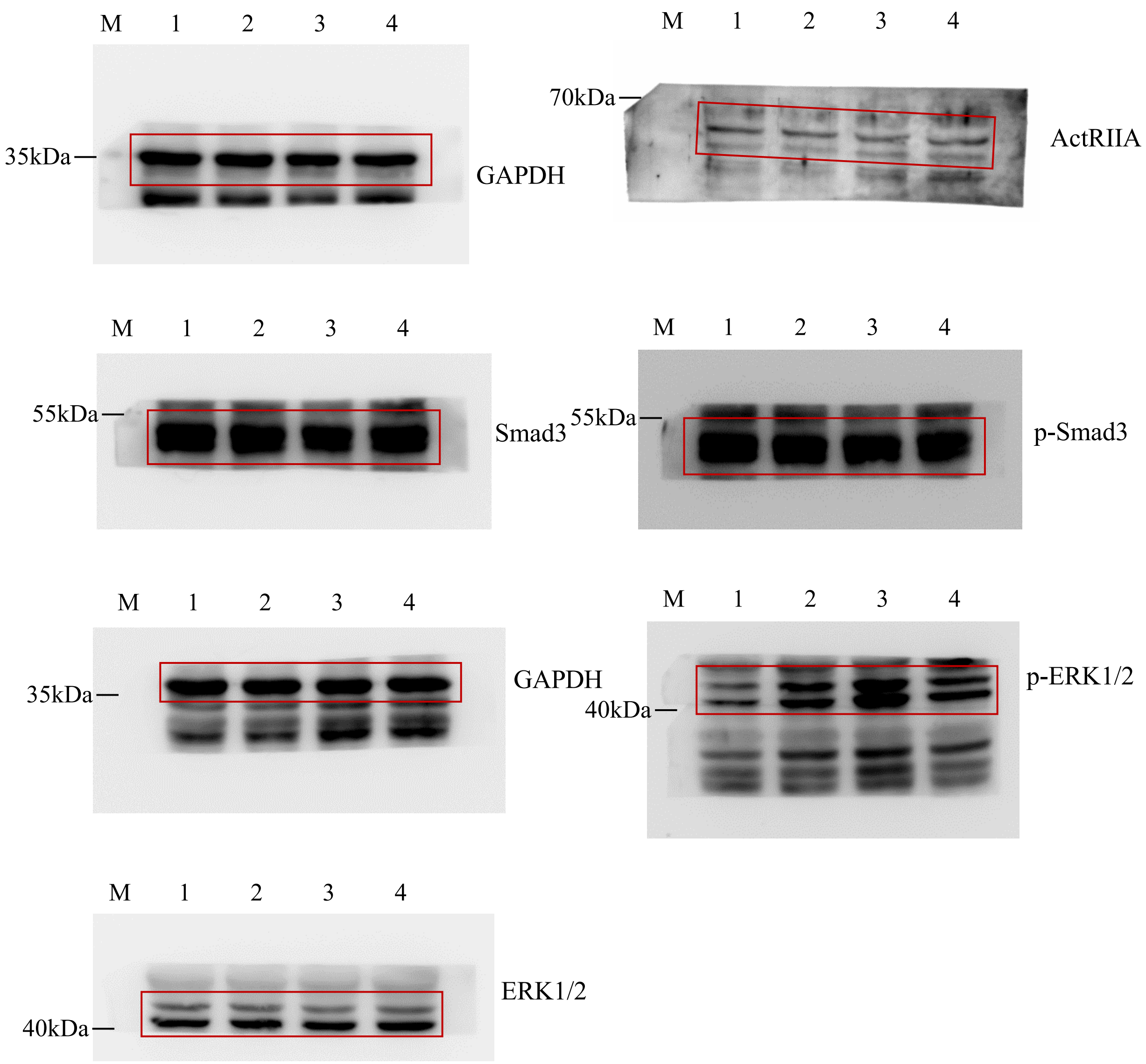

Supplementary Figure 8. Full-length blots of cropped images (red boxes) shown in manuscript Figure 7b and 7c. The membrane of full-length western blots was cut according to colorful molecular marker of protein, and then the cut membranes were probed with anti-GAPDH, ActRIIA, Smad3, p-Smad3, ERK1/2 and p-ERK1/2 antibodies respectively and the labeled proteins were detected by chemiluminescence. M, colorful molecular marker of protein. Lane 1, control; Lane 2, activin A (10 ng/ml); Lane 3, TNF-α (10 ng/ml); Lane 4, activin A (10 ng/ml) + TNF-α (10 ng/ml).

Supplementary Results

Effects of activin A and TNF-α on IL-6 production in HGFs

To analyze whether there was similar action of activin A and TNF-α in the HGFs as in mouse fibroblast L929 cells, the levels of IL-6 production from HGFs were examined by ELISA. As shown in Supp. Fig. 1a, the obtained HGFs was spindle or polygonal morphology, which was consistent with fibroblast-like morphology[1]. ELISA results showed that activin A had on significant effect on IL-6 secretion, while TNF-α remarkedly increased IL-6 release by HGFs, but co-treatment with activin A inhibited this effect (Supp. Fig. 1b), which were consistent with the results of IL-6 production in mouse L929 cells.

Effects of activin A and TNF-α on the viability of HGFs

It has been widely recognized that TNF-α inhibits mouse fibroblasts viability[2]. In this study, CCK-8 assay was used to examine the viability of HGFs treated with activin A and/or TNF-α. The data showed that TNF-α suppressed HGFs viability, while activin A alone did not affect the cell viability, but alleviated the inhibitory effect of TNF-α on cell viability (Supp. Fig. 2), which were consistent with the results of cell viability in mouse L929 cells. These findings indicate that activin A and TNF-α have similar effects on regulating the activity of mouse and human fibroblasts.

Supplementary Methods

Human gingival fibroblasts culture

HGFs were obtained from an explant culture of healthy human gingiva as described previously[3]. HGFs were cultured in DMEM supplemented with 10% fetal bovine

serum (FBS), penicillin (100 U/ml) and streptomycin (100 μg/ml) at 37 °C with 5% CO_2. Cells between passages 4 to 7 were used in this study. This research was approved by the ethical review committees of School and Hospital of Stomatology and College of Basic Medical Sciences, Jilin University. Written informed consent was provided from all participants and then sampling was performed. Samples of gingival tissue were harvested from healthy donors who underwent surgery to extract the lower third molar at the School and Hospital of Stomatology, Jilin University. All procedures were performed according to the current guidelines.

Cell viability assay

Cell viability was determined using CCK-8 assay. HGFs (5×10^3 cells per well) were seeded into a 96-well plate and incubated in 1% FBS-DMEM containing activin A (0, 5 and 10 ng/mL), TNF-α (5 and 10 ng/mL) and activin A (10 ng/mL) plus TNF-α (5 or 10 ng/mL) at 37°C in 5% CO_2 for 24 h, respectively. 10 μl of CCK-8 reagent was added into the culture medium of each well, and the cells were incubated for 2 h at 37°C. The absorbance was detected at 450 nm with a microplate spectrophotometer. Each experiment was carried out in triplicate.

Enzyme-linked immunosorbent assay

The supernatants of the cultured HGFs were collected and the levels of IL-6 were determined by ELISA kits according to the manufacturer’s protocol. The absorbance was then detected at 450 nm to evaluate the IL-6 levels using a microplate spectrophotometer.

Supplementary References

1. Wyganowska-Swiatkowska, M. *et al.* Clinical implications of the growth-suppressive effects of chlorhexidine at low and high concentrations on human gingival fibroblasts and changes in morphology. *Int. J. Mol. Med.* **37**, 1594-1600 (2016).
2. Lui, P. C. *et al.* Apoptotic and necrotic effects of tumour necrosis factor-alpha potentiated with hyperthermia on L929 and tumour necrosis factor-alpha-resistant L929. *Int. J. Hyperthermia* **26**, 556-564 (2010).
3. Zdarilová, A., Svobodová, A., Simánek, V. & Ulrichová, J. Prunella vulgaris extract and rosmarinic acid suppress lipopolysaccharide-induced alteration in human gingival fibroblasts. *Toxicol. In Vitro* **23**, 386-392 (2009).
